# Suprafamilial relationships among Rodentia and the phylogenetic effect of removing fast-evolving nucleotides in mitochondrial, exon and intron fragments

**DOI:** 10.1186/1471-2148-8-321

**Published:** 2008-11-26

**Authors:** Claudine Montgelard, Ellen Forty, Véronique Arnal, Conrad A Matthee

**Affiliations:** 1 Institut des Sciences de l'Evolution de Montpellier (UMR 5554), Université de Montpellier II, Place Eugène Bataillon, 34095 Montpellier cedex, France; 2 Evolutionary Genomics Group, Department of Botany and Zoology, Stellenbosch University, Private Bag X1, Matieland, Stellenbosch 7602, South Africa; 3 Current address : Biogéographie et Ecologie des Vertébrés (EPHE), Centre d'Ecologie Fonctionnelle et Evolutive (UMR 5175), 1919 Route de Mende, 34293 Montpellier cedex 5, France

## Abstract

**Background:**

The number of rodent clades identified above the family level is contentious, and to date, no consensus has been reached on the basal evolutionary relationships among all rodent families. Rodent suprafamilial phylogenetic relationships are investigated in the present study using ~7600 nucleotide characters derived from two mitochondrial genes (Cytochrome *b *and 12S rRNA), two nuclear exons (IRBP and vWF) and four nuclear introns (MGF, PRKC, SPTBN, THY). Because increasing the number of nucleotides does not necessarily increase phylogenetic signal (especially if the data is saturated), we assess the potential impact of saturation for each dataset by removing the fastest-evolving positions that have been recognized as sources of inconsistencies in phylogenetics.

**Results:**

Taxonomic sampling included multiple representatives of all five rodent suborders described. Fast-evolving positions for each dataset were identified individually using a discrete gamma rate category and sites belonging to the most rapidly evolving eighth gamma category were removed. Phylogenetic tree reconstructions were performed on individual and combined datasets using Parsimony, Bayesian, and partitioned Maximum Likelihood criteria. Removal of fast-evolving positions enhanced the phylogenetic signal to noise ratio but the improvement in resolution was not consistent across different data types. The results suggested that elimination of fastest sites only improved the support for nodes moderately affected by homoplasy (the deepest nodes for introns and more recent nodes for exons and mitochondrial genes).

**Conclusion:**

The present study based on eight DNA fragments supports a fully resolved higher level rodent phylogeny with moderate to significant nodal support. Two inter-suprafamilial associations emerged. The first comprised a monophyletic assemblage containing the Anomaluromorpha (Anomaluridae + Pedetidae) + Myomorpha (Muridae + Dipodidae) as sister clade to the Castorimorpha (Castoridae + Geomyoidea). The second suprafamilial clustering identified a novel association between the Sciuromorpha (Gliridae + (Sciuridae + Aplodontidae)) and the Hystricomorpha (Ctenodactylidae + Hystricognathi) which together represents the earliest dichotomy among Rodentia. Molecular time estimates using a relaxed Bayesian molecular clock dates the appearance of the five suborders nearly contemporaniously at the KT boundary and this is congruent with suggestions of an early explosion of rodent diversity. Based on these newly proposed phylogenetic relationships, the evolution of the zygomasseteric pattern that has been used for a long time in rodent systematics is evaluated.

## Background

Since the pioneer work of Brandt [1], a wealth of literature has been devoted to suprafamilial relationships among rodents. To date, however, no consensus has been reached based on morphological or paleontological evidence. Nearly a century after Brandt [1], Simpson ([2], p. 197) referred to the order Rodentia and stated that "their relationships are involved in an intricate web of convergence, divergence, parallelism, and other taxonomic pitfalls."

The addition of molecular data contributed significantly in constructing a species tree for the order Rodentia and the most up to date taxonomic arrangement includes at least 2277 species distributed among 33 families and five suborders [3]. Recently Huchon et al. [4] recognized the Laotian rock rat (*Laonastes aenigmamus*) from Laos [5] as an additional family Diatomyidae closely related to the Ctenodactylidae. Despite this new addition, the number of initially recognized rodent families by Simpson [2] and Wood [6] remained fairly stable (for review see [3]). The number of rodent clades identified above the familial level, however, led to numerous inconsistencies and controversies (see [7-9]). In the present study we adopted the most up to date suprafamilial classification as reviewed by Carleton and Musser [3] who recognize five suborders (Sciuromorpha, Castorimorpha, Myomorpha, Anomaluromorpha and Hystricomorpha).

Hystricomorpha contains 19 families (78 genera and 291 species), and includes the previously problematic Ctenodactylidae [3] and the newly discovered Diatomyidae [4]. The two latter families were identified as the sister taxon of the 17 traditional families comprising the infraorder Hystricognathi [4,10]. The monophyly of Hystricomorpha is currently supported by morphological, paleontological and molecular data (see review in [10-13]). Sciuromorpha includes Gliridae, Aplodontidae and Sciuridae. The latter two families are closely related based on hard and soft morphological features [14-17], albumin immunology [18] and sequence data (for example see [13,19-21]). The myomorphous Gliridae is regarded as an early offshoot of Sciuromorpha and this is supported by middle ear anatomy [14], arterial patterns [22]) and previous molecular investigations (for example [19,21,23]). Castorimorpha also comprises three families, Castoridae, Heteromyidae and Geomyidae. This association was first suggested by Tullberg [24] and, although not well supported by morphology, has fairly strong molecular support (for example see [13,19-21]). The two superfamilies, Dipodoidea and Muroidea (including one and six families, respectively) comprise the suborder Myomorpha and their close affinity is well established (see [3]). The Anomaluromorpha contains Anomaluridae and Pedetidae. Associations between the later two families are strongly supported by mitochondrial and nuclear data [4,11,21,25] and this agrees with Winge [26] and Tullberg [24]. However, a recent paper by Horner et al. [27] based on the coding regions of the mitochondrial genome disagrees with these suggestions and places Anomaluridae (Pedetidae was not included) as a sister taxon of Hystricognathi.

Evolutionary associations among these five suborders are not well resolved [3] and even the monophyly of the order has been questioned in the past based on mtDNA analyses [28,29]. The notion of paraphyly of the Rodentia, however, was short lived and never supported by morphology and more comprehensive genetic studies [13,20,30,31]. Based on available evidence, Carleton and Musser [3], suggested that Sciuromorpha, Myomorpha and Hystricomorpha are well established while the monophyly and/or phylogenetic position of Castorimorpha and Anomaluromorpha is less secure. Subsequent retroposed SINEs provided additional evidence for the monophyly of Myomorpha, Anomaluromorpha and Hystricomorpha whereas no SINE has been identified for Castorimorpha or Sciuromorpha. A clade including Myomorpha, Anomaluromorpha and Castorimorpha (the "mouse-related clade" as defined by Huchon et al. [20]) was also confirmed by several unique SINE insertions [11,32]. Unfortunately, no SINE has been found for any relationships among the three members of the "mouse-related clade" (Myomorpha, Anomaluromorpha and Castorimorpha). Finally the phylogenetic relationships among the three major rodent groups: Sciuromorpha, "mouse-related clade" and Hystricomorpha are as yet unresolved.

The introduction of phylogenomics and whole organism genome sequencing (thousands of nucleotides or amino acids), coupled to the use of probabilistic methods based on models of sequence evolution, implicitly led to the belief that inconsistency in tree reconstructions will soon be something of the past. However, it is clear now that increasing the number of nucleotides does not always solve incongruence in phylogenetics [33-35]. Even phylogenomic reconstructions can result in biases, and as a consequence, produce well supported incorrect tree topologies (for example [33]). In addition, gene tree reconstructions are based on numerous implicit assumptions that are seldom tested (for example gene orthology, reversible time homogeneous substitution process, stationarity of base composition through time). Violations of these assumptions may lead to compositional bias, contrasted patterns of saturation and heterogeneous evolutionary rates among genes and lineages. Current phylogenetic reconstruction methods do not efficiently test and account for such biases, the consequence being reconstruction artefacts such as long branch attraction (see for example [36-38]). To avoid these pitfalls, some authors [34,37,39] emphasize the necessity to test the quality and consistency of the data and recommended that sources of inconsistencies should be excluded (such as fast-evolving or compositionally biased positions). This is more feasible with large datasets because removing a part of the data will theoretically leave enough informative positions to recover confidence and consistency.

The aims of this paper are firstly to test the current phylogenetic hypotheses surrounding the higher level relationships among rodent families. Moreover, by using a large dataset we hoped to decipher remaining unsolved relationships among the five recognized rodent suborders. Secondly, we were particularly interested in comparing the contribution of three different datasets: two mitochondrial genes (Cytochrome *b *and 12S rRNA), two nuclear exons (the exon 28 of von Willebrand factor – vWF; exon one of the interphotoreceptor retinoid-binding protein – IRBP) and four nuclear introns (Stem cell factor – MGF; protein kinase C – PRKC; β-spectrin non erythrocytic 1 – SPTBN; and Thyrothropin-THY). For each dataset, we determined the distribution of sites according to eight evolutionary rates and we documented how the removal of the fast-evolving positions influenced phylogenetic reconstructions.

## Results

### Alignment, partition and heterogeneity of substitution rates

The alignments of the mitochondrial cyt*b *and 12S rRNA genes are respectively 1140 bp and 1042 bp long. A total of 56 bp in a loop region could not be aligned for the 12S rRNA fragment and was excluded (positions 933–987). The mitochondrial dataset comprised 2126 bp and was subdivided into 5 partitions: one for each codon position of cyt*b *(380 bp each), and stems (458 bp) and loops (528 bp) for the 12S rRNA region. The two nuclear exons, IRBP and vWF represented 1299 bp and 1272 bp respectively. The resulting 2571 positions have been partitioned into the three codon positions either for each gene separately (3 partitions of 433 bp each for IRBP and of 424 bp each for vWF) or from the 2 genes concatenated (3 partitions of 857 bp each). For the introns (MGF, PRKC, SPTBN and THY), the number of base pairs for the full alignments and those remaining after removal of the poorly aligned positions with Gblocks, together with the number of positions in intronic and exonic regions, are indicated for each gene in Table 1 (also see Additional files 1 and 2 for intron alignment before and after Gblocks). Although the total length of each intron varied considerably between taxa (Table 1), the number of conserved positions used for phylogeny reconstruction was close to the mean length for each fragment. For each gene and each pair of taxa, we graphically compared the p-distances (percent divergence) before and after removal of poorly aligned positions using Gblocks. With the exception of PRKC, the slopes of the regression lines (MGF: 0.89, PRKC: 0.62, SPTBN: 0.83, THY: 0.76) indicated a fairly good correlation before and after the exclusion of poorly aligned regions.

**Table 1:** Intron sequences

	Total alignment	Conserved positions	Exon	Intron	Mean Intron Length	Standard deviation
MGF	1330	820	35	785	684	82
PRKC	2355	533	77	456	553	182
SPTBN	2578	833	77	756	706	159
THY	1790	711	227	484	481	91
TOTAL	8053	2897	416	2481	-	-

Number of positions: in the full alignment (column 1), after elimination of poorly aligned positions by Gblocks (column 2), in the remaining exonic parts (column 3) and in the intronic regions (column 4). For each gene, columns 5 and 6 give the mean intron length before alignment and its standard deviation.

The estimated number of sites in each of the eight gamma rate categories for the three main data types (mitochondrial, exon and intron data) is presented in Table 2. Using TREE-PUZZLE the proportion of invariable sites has been estimated to be zero in each case. Thus, invariable positions are all included in the first gamma rate category which encompasses the most sites for the three datasets, especially for the mitochondrial and exon genes (nearly 40% of sites). These latter two datasets show nearly no sites in the rate categories 2 and 3 (0 for mitochondrial genes and 31 for exons) whereas introns show a noticeable homogeneous increase between categories 2 to 7 (between 7.9% and 12.9% of sites). Fastest-evolving sites (category 8) are more numerous for introns when compared to the other two data types (exon and mtDNA). These results indicate that mitochondrial and exonic regions show a similar behaviour in terms of gamma rate distributions and vary greatly among sites: ~40% of the positions were invariable and ~12% reached a very high rate (5.42 and 3.91 for mitochondrial and exon genes, respectively). This heterogeneity is also evidenced in the gamma value of the distribution parameter alpha which varies from 0.20, 0.46 and 2.63 for mitochondrial, exon and intron datasets, respectively. The differences between the fragments sequenced can best be explained by the coding nature of mitochondrial and exon genes when compared to the non-coding introns.

**Table 2:** Gamma rate distribution for the mitochondrial (mito), exon and intron genes

Rate	MITO		EXON		INTRON	
Category	2126 sites	Rate	2571 sites	Rate	2897 sites	Rate
1	826 (38.8%)	0.000	944 (36.7%)	0.004	708 (24.4%)	0.275
2	0 (0%)	0.0006	0 (0%)	0.045	231 (8%)	0.475
3	0 (0%)	0.009	31 (1.2%)	0.144	228 (7.9%)	0.641
4	280 (13.2%)	0.052	371 (14.4%)	0.321	266 (9.2%)	0.806
5	228 (10.7%)	0.200	201 (7.8%)	0.607	241 (8.3%)	0.988
6	340 (16%)	0.614	347 (13.5%)	1.070	313 (10.8%)	1.207
7	187 (8.9%)	1.706	364 (14.2%)	1.890	374 (12.9%)	1.510
8	265 (12.4%)	5.420	313 (12.2%)	3.919	536 (18.5%)	2.10

For each dataset and the eight gamma categories, the number of sites (percentages in parentheses) is given in the left column and relative rates in the right column.

For the mitochondrial genes, 265 positions have been identified as fast-evolving sites (eighth relative gamma rate of 5.42) and subsequently removed. For the cyt*b *gene, 157 positions were eliminated (see Table 3) of which 135 were at third codon position whereas only one of the removed characters was at a second codon position. Stems and loops of the 12S rRNA gene are also markedly different with 103 of the 108 positions excluded occurring in the loop section. As for the coding-cyt*b*, exclusion of fast-evolving positions for the two concatenated exons (IRBP and vWF) was also concentrated at third codon positions (246 third position sites out of 312 excluded; eighth gamma rate of 3.92). For introns, 536 fast-evolving positions (eighth gamma rate of 2.1) were excluded representing 149 sites for MGF, 91 for PRKC, 181 for SPTBN, 97 for THY and 18 for the combined flanking-exonic regions of the introns. When sites corresponding to the eighth gamma category are removed, amplitude of evolutionary rates becomes 0.0001–4.80 (α = 0.28) for mitochondrial genes, 0.012–3.56 (α = 0.59) for exons and 0.51–1.63 (α = 7.16) for introns. In terms of heterogeneity of substitution rates, improvement is substantial for introns (α comes from 2.63 to 7.16) but much less for exons (0.46 to 0.59) and mitochondrial genes (0.20 to 0.28).

**Table 3:** Slope of saturation for each gene partition before and after (in italics) removal of fast-evolving positions

		Slopes of saturation (number of position considered)		
Gene	Total number of position	Partition 1	Partition 2	Partition 3

Cytochrome *b*	1140 *983 (86%)*	0.13 (380) *0.21 (359)*	0.42 (380) *0.43 (379)*	0.009 (380) *0.09 (245)*
12S rRNA	986 *878 (89%)*	0.29 (458) *0.33 (453)*	0.12 (528) *0.20 (425)*	
IRBP	1272 *1148 (90%)*	0.55 (433) *0.57 (421)*	0.59 (433) *0.77 (413)*	0.25 (433) *0.32 (314)*
vWF	1299 *1110 (85%)*	0.52 (424) *0.63 (406)*	0.55 (424) *0.60 (407)*	0.11 (424) *0.20 (297)*
MGF	785 *636 (81%)*	0.70 *0.88*		
PRKC	456 *365 (80%)*	0.56 *0.69*		
SPTBN	756 *575 (76%)*	0.65 *0.86*		
THY	484 *387 (80%)*	0.58 *0.72*		
Flanking-Exons of introns	416 *398 (96%)*	0.31 *0.43*		

Protein coding genes (cytochrome *b*, vWF and IRBP) have been partitioned according to codon positions, the 12S rRNA is partitioned in stems and loops and there is one partition for each intron and one partition for combined exons. In each case, the number of positions is given in parentheses.

### Base composition and saturation analysis

For each dataset (introns, exons and mitochondrial genes), several taxa deviate significantly in base composition when compared to the average base frequencies of the total alignment calculated by TREE-PUZZLE. For introns, eight out of 30 rodents deviate from the average composition. When fast-evolving sites are removed (18.5% of the alignment; see Table 2), deviation in base composition was confined to six taxa (*Mus*, *Geomys*, *Heteromys*, *Dipodomys*, *Cavia*, *Hystrix*). The exons (IRBP and vWF) and mitochondrial regions showed respectively 12 and 8 taxa (out of 29 rodents; see Additional file 3) deviating in base compositions. After removing 12% of the fast evolving positions in the exons and also in the mitochondrial regions, only one (*Spalax*) and three (*Heteromys*, *Pedetes*, *Mesocricetus*) taxa showed base composition deviations. It can be concluded that the fastest-evolving positions are partly responsible for the biases in composition and it seems reasonable to suggest that the exclusion of some of these biases will reduce the violations associated with base composition assumptions. It can also be noted, however, that in all datasets, taxa deviating in base composition were found to cluster at their expected phylogenetic position (before and after removal of fastest sites).

Saturation was estimated for each partition, before and after removal of fast-evolving sites. When using complete sequences, the slopes of the linear regressions (Table 3) indicated that 4 partitions in particular appeared saturated (S < 0.13): first and third codon positions of cyt*b*, loops in 12S rRNA and third codon positions of vWF. Third positions of IRBP, stems of the 12S rRNA and the flanking-exons of introns are moderately saturated (S = 0.25, 0.29 and 0.31, respectively). The nine remaining partitions (mostly confined to intronic regions) are least saturated and probably also the most informative phylogenetically (S > 0.42). Removal of fast-evolving positions improves the phylogenetic signal, as indicated by the steeper slope values for the 16 partitions tested (Table 3). For third codon positions of cyt*b*, the slope is increased by an order of magnitude of 10, even though the resulting value (0.09) is still indicative of significant saturation present at this position. As shown previously (for example see [40,41]), the mitochondrial dataset is the most saturated whereas the nuclear genes (exons and introns) are less affected. Our analyses demonstrated that removal of the fastest evolving sites decrease saturation in the data and, although we believe that this provides a substantial improvement, saturation could not be totally eliminated.

### Contribution of different data types to rodent phylogenetics

**Figure 1:**
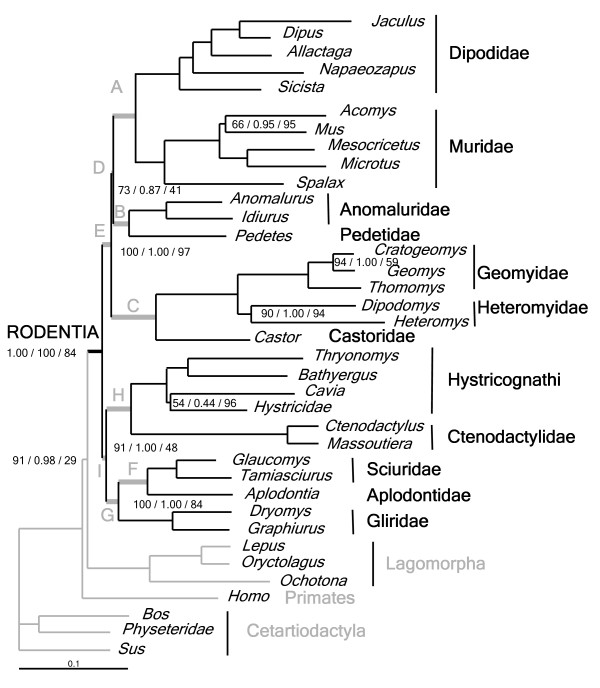
**Bayesian phylogram of Rodentia**. Phylogenetic relationships are inferred from the reduced-concatenated dataset. Numbers at nodes refer, from left to right respectively, to bootstrap percentages in ML analysis with RAxML (100 replications), to posterior probabilities in Bayesian analysis and to bootstrap percentages in MP analysis with PAUP (1000 replications). Only nodes not supported by posterior probabilities of 1.00 or 100% BP are indicated. In both probabilistic analyses, dataset was analysed with the GTR + I + G model applied to 13 independent partitions (see text for details). Rodent family names are indicated on the right and grey upper case letters at nodes correspond to suprafamilial groupings as defined in Additional file 4.

The various analyses performed in the present study supported the monophyly of all rodent families represented by two or more taxa (see Additional file 3). For each dataset (mitochondrial, exon and intron), nodal support obtained from the MLP (partitioned maximum likelihood) and Bayesian analyses is provided as Additional file 4 for different suprafamilial groupings (letters correspond to clades labelled on Figure 1). When the different datasets are compared, only 4 clades are supported by all three types of data separately: A-Myomorpha, B-Anomaluromorpha, F-Sciuroidea and H-Hystricomorpha. Two groupings (C-Castorimorpha and E-Myomorpha + Anomaluromorpha + Castorimorpha) are weakly supported by mitochondrial genes and moderately by exons whereas clade G-Sciuromorpha received the most support from the mitochondrial dataset. By comparison, the intronic regions that are less affected by bias in rate distributions among sites, and seem to be less saturated, gave more resolution than the exon or mitochondrial data sets. Well established clades from the literature are strongly supported and moreover the introns also suggest two other subordinal relationships. First, inside the "mouse-related clade" (E-Castorimorpha + Myomorpha + Anomaluromorpha), the Myomorpha cluster with Anomaluromorpha (BP = 100, BI = 1.00) to the exclusion of the 2 other alternatives (D2 and D3 in Additional file 4). Secondly, the introns suggest a less secure but consistent sister taxon relationship between Sciuromorpha and Hystricomorpha (BP = 54, BI = 0.69). The contribution of each intron to this node is mixed since PRKC (BI = 0.94) and THY (BI = 0.64) support this grouping while MGF (BI = 0.82) and SPTBN (BI = 0.96) rather suggest a basal split for Hystricomorpha as the first emerging rodent clade. On the other hand, all four introns individually found Anomaluromorpha as sister group to Myomorpha (BI = 0.46 to 0.85). It is noticeable that separate analyses of the introns each contributed signal for these difficult nodes whereas each mitochondrial and exonic gene does not suggest any relationships.

Removal of fast-evolving positions leads to mixed results. For the mitochondrial and exon genes, there is a clear improvement for the support for three clades: C-Castorimorpha, E-(Myomorpha+Anomaluromorpha) + Castorimorpha and G-Sciuromorpha. With the mitochondrial dataset, however, the well established Anomaluromorpha and Myomorpha clades [4,21] are distorted when characters are excluded because of the inclusion of *Jaculus *as a sister species of *Anomalurus*. When taken separately, the exclusion of fast evolving sites for introns negatively affected the support for most nodes (data not shown), whereas, when concatenated, all of them (to the exception of the grouping D-Myomorpha+Anomaluromorpha) are strongly recovered (see Table 4 and Additional file 4). Furthermore, a noticeable increase in support was found for Sciuromorpha as a sister clade to Hystricomorpha (BP = 95, BI = 1.00) at the base of the tree.

**Table 4:** Supports for two suprafamilial groupings according to various datasets: the three separate (mitochondrial, exon and intron genes), the three combinations of two datasets and all genes concatenated (conc).

	Myomorpha + Castorimorpha + Anomaluromorpha			Sciuromorpha + Hystricomorpha + "Mouse-related" clade		
	
	Myo + Ano	Myo + Casto	Casto + Ano	Sciuro + Hystrico	Hystrico basal	Sciuro basal
MITO (2126)	10/0.17	14/0.37	15/0.39	5/-	-/-	3/0.48
R-MITO (1861)	-/-	2/-	9/-	29/-	4/0.33	2/-

EXON (2571)	38/-	29/0.70	-/0.05	5/0.08	37/0.74	16/-
R-EXON (2258)	31/0.7	16/0.2	1/0.09	17/0.09	57/0.81	11/0.08

INTRON (2897)	100/1.00	-/-	-/-	54/0.69	30/0.28	16/-
R-INTRON (2361)	77/0.79	20/0.14	3/0.08	95/1.00	-/-	5/-

MITO + EXON (4697)	32/0.17	56/0.74	7/0.09	4/-	26/-	54/0.99
R-MITO + R-EXON (4119)	5/-	-/-	-/-	30/0.16	46/0.57	14/0.27

MITO + INTRON (5023)	95/0.99	3/-	2/-	37/0.43	26/0.13	37/0.45
R-MITO + R-INTRON (4222)	74/0.70	26/0.21	-/0.08	98/1.00	-/-	2/-

EXON + INTRON (5468)	90/0.97	10/-	-/-	44/0.38	46/0.57	10/-
R-EXON + R-INTRON (4619)	84/0.92	15/0.06	1/-	90/1.00	7/-	5/-

CONC (7594)	88/0.93	12/0.06	27/0.07	37/0.23	36/0.32	27/0.45
R-CONC (6480)	73/0.87	-/-	-/-	91/1.00	5/-	4/-

Each dataset is analysed with and without (noted R for reduced) fast-evolving sites and the numbers of characters analysed is indicated in parenthesis. In each case, the bootstrap support resulting from 100 replications in partitioned maximum likelihood analysis with RaxML and the posterior probability in Bayesian analysis with MrBayes are indicated from right to left, respectively for the three possible relationships among the "mouse-related clade" (Myomorpha, Castorimorpha and Anomaluromorpha) and between the three main rodent lineages (Sciuromorpha, Hystricomorpha and "Mouse-related" clade).

For mitochondrial and exon datasets, we further exclude potential homoplasious characters by eliminating fast-evolving sites belonging to the Gamma rate category 7. A total of 187 additional positions were eliminated for the whole mitochondrial dataset. For exons, 223 sites were additionally removed at the third positions only because saturation analyses revealed that first and second positions were not plagued by saturation after elimination of rate category 8 (see Table 3). Thus, 1674 and 2035 positions were reanalysed for the mitochondrial and exon datasets, respectively. Analysis with PUZZLE indicated no improvement in among site rate variation with the intervals ranging between 0.0001–4.68 (α = 0.29) for mitochondrial genes and 0.0165–3.46 (α = 0.64) for exons. Phylogenetic analyses conducted with RAxML on these reduced datasets only led to the deterioration of support for various phylogenetic relationships and in fact rather found more ambiguous clusterings (an unlikely phylogenetic position was found for *Castor*, *Pedetes *and *Homo*). The exclusion of these data thus clearly reflect a decrease in the resolving power of the data and therefore support suggestions that more saturated data also contains phylogenetic signal [42,43]. The same explanation can also be put forward to explain the reduced support for the Myomorpha + Anomaluromorpha node after removal of fastest sites (see Table 4).

Finally, to further explore the utility of each dataset (mitochondrial genes, exons and introns) the three datasets (with and without fast-evolving sites) were combined in a pairwise fashion (Table 4) and results are presented for the two main nodes of interest (relationships among the "mouse-related clade" and between the three main rodent lineages). Based on all nucleotides, none of the three pairwise combinations support the branching pattern between the three main rodent clades. After removal of the fastest-evolving positions, the clade Hystricomorpha + Sciuromorpha is supported by two out of three combinations (Table 4). In fact, the combined mitochondrial genes + exons do not support any one of the two clades. On the contrary, the combinations that included the intron data were fully congruent with the combined analyses in the sense that the clade Anomaluromorpha + Myomorpha is well supported and the Sciuromorpha + Hystricomorpha is revealed after elimination of fastest positions.

### Concatenation of datasets, alternative hypotheses and molecular dating

Concatenation of the eight genes resulted in the analyses of 7594 characters for the full dataset and 6480 characters when fast-evolving sites are removed. Results are presented in Table 4, Figure 1, and Additional file 4. With the two probabilistic approaches, removal of fast-evolving sites recovered a strong basal clade uniting Hystricomorpha+Sciuromorpha (BP = 91, BI = 1.00; clade I in Additional file 4) whereas less support for this grouping was obtained using the full data set (BP = 37, BI = 0.22 for the same clade). The "mouse-related group" (clade E) is strongly supported in both cases and the sister taxon relationship between Myomorpha and Anomaluromorpha (clade D) is well supported by both data treatments (reduced dataset: BP = 73, BI = 0.90; all data: BP = 88, BI = 0.93). The remaining rodent relationships also received good support when using concatenated gene sequences and confirmed an increase in phylogenetic resolution when data are combined (see for example [44-47]).

For the MP analyses, the number of informative characters was 4219 and 3595, for the complete and reduced datasets respectively. Only one tree was recovered in each case and, as with probabilistic methods, most relationships were strongly supported (see Additional file 4). The two parsimony trees differed in the basal branching order in that the complete dataset suggests the sister group relationship between Sciuromorpha and the "mouse-related clade" (group I2 in Additional file 4; BP = 78) whereas the reduced dataset weakly supports the clustering Sciuromorpha + Hystricomorpha (clade I; BP = 48). As with other reconstruction methods, the clade Myomorpha+Anomaluromorpha (clade D) is better supported by the complete (BP = 72) than by the reduced (BP = 41) dataset.

When the 1113 fastest evolving sites (that were excluded from the analyses above) were analysed separately, (100 bootstrap replications with PHYML; data not shown) the well supported relationships such as the monophyly of the five rodent suborders was supported (moderately for Sciuromorpha: BP = 55; and stronger for the other four clades A, B, C and H in Additional file 4: BP range 82–99). At the higher level clade E-(Myomorpha + Anomaluromorpha) + Castorimorpha was found (BP = 82), but other relationships (Myomorpha + Castorimorpha and Hystricomorpha as the first emergence in Rodentia) were weakly supported (BP = 43 and 42, respectively).

To evaluate the stability of the most likely topology (Figure 1), we tested nine hypothetical topologies representing the clustering possibilities between suborders of "E-mouse-related clade" (A-Myomorpha, B-Anomaluromorpha and C-Castorimorpha), and "G-Sciuromorpha" and "H-Hystricomorpha". When these nine topologies are evaluated on the whole concatenated dataset, results of the AU and SH tests indicated that tree-1 is identified as the best hypothesis but none of the other eight topologies were significantly worse (at the 5% level) than the most likely tree (Table 5). Both tests are congruent even if probabilities obtained are sometimes quite different (see hypotheses 5 and 9). After removal of fast-evolving sites, tree-1 is still identified as the best topology and P-values increased. Five out the nine trees (5 to 9 in Table 5) can reasonably be rejected and the grouping I-Sciuromorpha+Hystricomorpha is consistently supported. Posterior probabilities also decreased for hypotheses indicated by trees 4 to 9. However, trees 2 and 3 are also not supported by PP when the whole dataset is tested.

**Table 5:** Three tests of nine *a priori *topologies

Topology tested	Whole Dataset 7594 nucleotides			Reduced Dataset 6480 nucleotides		
	**AU**	**SH**	**PP**	**AU**	**SH**	**PP**

**1 ((((Myo, Ano), Casto), (Hyst, Sciu))**	**0.670**	**0.910**	**0.539**	**0.853**	**0.92**	**0.691**
2 (((((Casto, Ano), Myo), (Hyst, Sciu))	0.126	0.580	0.018	0.432	0.662	0.196
3 ((((Myo, Casto), Ano), (Hyst, Sciu))	0.315	0.615	0.028	0.211	0.591	0.111
4 ((((Myo, Ano), Casto), Sciu), Hyst)	0.544	0.799	0.215	0.176	0.149	0.001
5 ((((Casto, Ano), Myo), Sciu), Hyst)	0.056	0.463	0.005	0.066	0.083	<10-3
6 ((((Myo, Casto), Ano), Sciu), Hyst)	0.297	0.561	0.014	0.049	0.059	<10-5
7 ((((Myo, Casto), Ano), Hyst,) Sciu)	0.296	0.540	0.011	0.051	0.079	10-4
8 ((((Myo, Ano), Casto), Hyst,) Sciu)	0.540	0.778	0.165	0.044	0.128	0.001
9 ((((Casto, Ano), Myo), Hyst,) Sciu)	0.093	0.464	0.005	0.035	0.066	<10-6

P-values of the approximately unbiased test (AU), Shimodaira-Hasegawa test (SH) and approximate Bayesian posterior probability (PP) for nine topologies tested before (whole) and after (reduced) removal of fast-evolving positions. The best topology is indicated in bold with the following abbreviations: Myo = Myomorpha; Ano = Anomaluromorpha; Casto = Castorimorpha; Hyst = Hystricomorpha; Sciu = Sciuromorpha.

**Figure 2:**
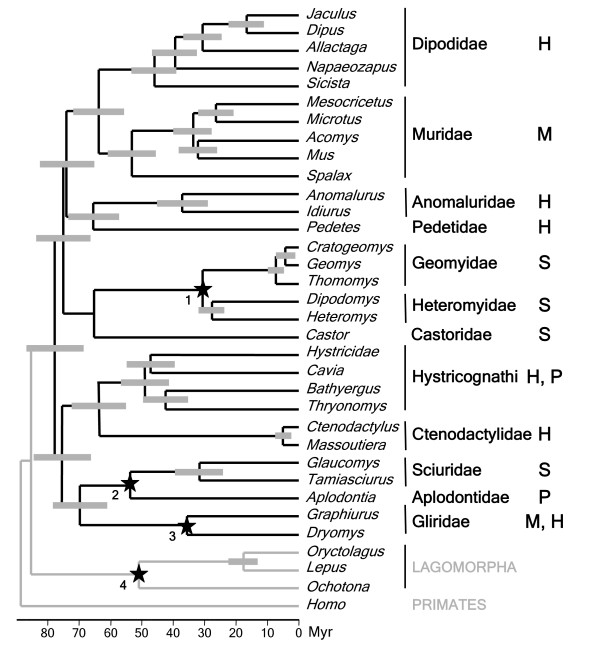
**Chronogram of rodent diversification**. Divergence times are calculated from the reduced-concatenated dataset using a relaxed molecular clock with four constrained nodes numbered 1 to 4 (see text for details) and represented by stars. Estimated dates with the 95% credibility intervals are represented by grey boxes at nodes. Time scale is indicated in Million of years below the figure. The zygomasseteric condition (H: Hystricomorphy; M: Myomorphy; S: Sciuromorphy; P: Protogomorphy) of each rodent family is specified on the right.

Estimation of divergence times and the 95% credibility intervals are reported for each clade on the chronogram in Figure 2. The Ctenodactylidae and Geomyidae families show a recent origin: around 5 and 7 Mya (95% interval 3.1–7.6 and 5.1–10.5 Mya), whereas Dipodidae is the oldest family originating approximately 47 Mya (40.3–54.6 Mya). Other families (Cricetidae, Heteromyidae, Sciuridae, Muridae, Gliridae, and Anomaluridae) originated between 27 and 38 Mya (95% interval between 27.1 and 46.5 Mya). Four of the five suborders (Hystricomorpha, Myomorpha, Castorimorpha, and Anomaluromorpha) diversified nearly contemporaneously between 65 and 67 Mya (interval between 57.2 and 75.7 Mya) whereas Sciuromorpha diverged earlier at 71.5 Mya (63.4–80.5 Mya). The deepest bifurcations among rodents (Sciuromorpha+Hystricomorpha, the "mouse-related clade" and Myomorpha+Anomaluromorpha) are dated to around 77 Mya (67.9–86.8 Mya) and the origin of order Rodentia is estimated around 79.8 Mya (71.4–89.2 Mya).

## Discussion

### Removal of fast-evolving sites and contribution to rodent phylogeny

The objective of removing fast-evolving positions was first to identify and improve the signal to noise ratio in all three different datasets (mitochondrial, exon and intron fragments) that showed different patterns of evolutionary rates. The first conclusion we reached, in agreement with Rodriguez-Espelata et al. [37], is that fast-evolving sites are positively correlated with saturation and these sites also suffer the most from compositional bias. In most instances the elimination of these sites resulted in better supported relationships among rodent suborders. There was also an indirect indication of an increase in the phylogenetic signal for all partitions tested, as measured by base composition and saturation analyses (Table 3). However, the indiscriminate removal of fast evolving sites actually decreased the phylogenetic resolution in some instances (for example when both categories 7 and 8 were removed).

We observed that the proportion of fastest sites is greater in introns than in the other two data sets (18.5% for introns *vs *12% for exon and mitochondrial genes) as the non-coding introns are under less selection. Nonetheless, removing of fast-evolving positions had little impact on gamma rate distribution (Table 2) and also the global heterogeneity as measured by the alpha parameter. This result is not really surprising because mitochondrial and exonic regions are characterized by much contrasted categories among sites with numerous positions (nearly 40%) that do not vary (rate category 1). Removal of the few fastest positions (rate category 8) does not really influence the overall distribution. A more uniform distribution of evolutionary rates is one reason for making introns valuable evolutionary markers (see also [48]) especially when compared to mitochondrial and exonic genes which encompass a big proportion of invariable sites alternating with a relatively large proportion of fast-evolving positions. These categories are either useless (invariable sites) or problematic (homoplasy in fast-evolving sites) for reconstructing phylogenetic relationships.

The removal of the fast-evolving positions improved support for a number of nodes but at different taxonomic levels. For introns, the reduced dataset improved the basal split among rodent suborders (node I in Additional file 4) whereas for mitochondrial and exonic regions, support is increased at the more terminal nodes (C, E or G in Additional file 4). Our interpretation is that removing the fastest positions cannot totally eliminate saturation (see Table 3) and thus, elimination of fast-evolving sites can improve the support only for nodes that are moderately affected by homoplasy (in our data set the deepest node for introns and more recent nodes for exons and mitochondrial genes).

With the two probabilistic methods of tree reconstruction, no substantial changes in the topology were observed between the whole and reduced concatenated datasets. Pisani [39] suggested that more sophisticated and realistic models of evolution can lead to a more robust topology. With MP analyses, the complete matrix suggests a grouping Sciuromorpha + "mouse-like clade" whereas the reduced dataset clustered Hystricomorpha with Sciuromorpha, which corresponds to the topology obtained using ML and BI reconstructions. Following the arguments proposed by Bergsten [49], these conflicting topologies might suggest that the MP tree (observed with the whole dataset) could result from long branch attraction as this "artefact" disappears when fast-evolving sites are removed. Strikingly, the reduced datasets contributed to a significant improvement when testing alternative topologies (see Table 5). Elimination of some noise in the data leads to better discrimination between the different topologies.

Our conclusion is that identification and removal of fast-evolving positions has been shown to be useful in revealing some phylogenetic information previously concealed by homoplasy [34]. Moreover, elimination of a small number of sites (12–18% for our three datasets), particularly for introns and concatenation of markers, allows for increase in the support for deeper nodes. This method can effectively be useful because the deepest phylogenetic relationships, characterized by short internal branches, are very often the most difficult to resolve. Our recommendation would be that complete and reduced analyses should be conducted on the same dataset, in order to empirically confirm the presence and location of the phylogenetic signal.

### Early rodent relationships and evolution of Rodentia

This study fully support the recognition of the five subordinal clades as described in Carleton and Musser [3] and previously identified in several molecular studies [4,11,13,19-21]. In addition to these five suborders, the "mouse-related clade" (E-Anomaluromorpha + Myomorpha + Castorimorpha), is strongly supported by the introns and the concatenated datasets with and without fastest sites and the support for this clade is reinforced with the reduced exon and mitochondrial datasets (see Table 4). Among this grouping, Anomaluromorpha is the sister taxon of Myomorpha, leaving Castorimorpha as the first offshoot in the "mouse-related clade". This branching order is strongly supported by the complete intron dataset and is only moderately supported by the reduced introns or by the complete- or reduced-concatenated datasets. Moreover, we found no indication of a basal position for Anomaluridae as suggested by Horner et al. [27]. Interestingly, when 25% of fastest evolving mtDNA amino acid sites were removed by Horner et al. [27] the Anomaluridae was placed as the sister taxa of Myomorpha. It is possible that the mtDNA result, placing the Anomaluridae at different positions, may be due to incomplete taxonomic sampling (only *Anomalurus *was inlcuded in the mtDNA study). Analyses of the two mtDNA genes included in the present study indeed reveal Anomaluromorpha included in the "mouse-related clade" (clade E in Additional file 4). The close phylogenetic association between Myomorpha and Anomaluromorpha is also strongly supported in Huchon et al. [4] and moderately supported in the papers of Adkins et al. [19] and Waddell and Shelley [13].

Our study provides the first evidence for a monophyletic clade comprising Hystricomorpha and Sciuromorpha and also the first evidence that this clade represents the deepest dichotomy amongst Rodentia. This association is mostly obtained by the intron data and also the combined analyses. When datasets are combined in a pairwise fashion, no significant conflicting phylogenetic signal was found between topologies derived from introns and those derived from the other two datasets (especially after removal of fast-evolving positions – see Table 4). Moreover, this clustering gained additional support when alternative hypotheses were compared (Table 5). Although this clade has never been proposed based on morphological or paleontological data it has been mentioned in previous molecular studies that were mostly based on limited taxonomic sampling for rodents [50-53]. Sciuromorpha as the first emergence among Rodentia represents an alternative hypothesis [13,19,54] but these studies were also based on limited taxonomic sampling. Finally, SINE data derived from two studies [11,32] could also not conclusively resolve the basal diversifications of rodents. Taken the data at hand, the early rodent dichotomies are complicated as also depicted by the short internal branches at the base of the tree. Two of our intron data sets gave good support for the monophyly of Hystricomorpha + Sciuromorpha while the two others suggest a basal position for Hystricomorpha as the first diverging rodent lineage. Considering these conflicts, we cannot rule out the possibility that the difference in branching order is a result of independent lineage sorting [55]. Although there is no strong phylogenetic conflict between the intron, exon and mitochondrial datasets (especially after removal of fast-evolving sites), resolving the basal node in Rodentia will require more data.

According to our molecular dating, the order Rodentia arose during the Late Cretaceous (65–99 Mya) between 71.4 and 89.2 Mya, which places its oldest origin before the KT boundary. This date is slightly older but comparable to the ranges suggested by Springer et al. [56] and Huchon et al. [4]. All these molecular estimations predate the oldest rodent fossils which are identified in the Late Paleocene (54.8–61 Mya; [57]) and are more in agreement with a Late Cretaceous superordinal diversification of placentals [58]. As soon as the early Eocene (49–54.8 Mya), Rodentia already appeared to be diverse and was present on all continents with the exception of South America [8]. Our date estimates are compatible with an early contemporaneous explosion of rodent diversity (roughly at the KT boundary) that gave rise to the five suborders (Myomorpha, Hystricomorpha, Anomaluromorpha, Castorimorpha, and Sciuromorpha).

One of the earliest classifications of rodents was proposed by Brandt [1] on different arrangements of the jaw musculature. Three types were recognized: sciuromorphy, myomorphy, hystricomorphy and Wood [6] added a fourth type: protrogomorphy. These morphotypes have been recognized as homoplasious for a long time [2,6,16,59]. For example, Marivaux et al. [12] came to the conclusion that the hystricomorphous condition arose at least four times independently. Based on our rodent phylogeny (Figure 2), it can be argued that these complex patterns are not entirely homoplasious. Sciuromorphy evolved merely twice (once in Sciuridae and once in Castorimorpha) but it is noteworthy that the four zygommasseteric arrangements found in the Sciuromorpha clade is an unique case among rodents since other major suprafamilial groupings are characterized by one or two types at most. Further detailed morphological or morphometric analyses could now be conducted to test if a pattern shared by several related rodent families (such as for example the hystricomorph condition of Pedetidae, Anomaluridae and Dipodidae) might be considered as real homology or if this pattern is only reflecting morphological grades (adaptation) without any phylogenetic meaning.

## Conclusion

Suprafamilial phylogenetic relationships among Rodentia were assessed using ~7600 characters including mitochondrial as well as exon and intron nuclear DNA. For each dataset, we determined the distribution of sites according to eight evolutionary Gamma rates and we assess the impact of removing fastest sites on phylogenetic reconstructions. Our conclusion is that fast-evolving sites are positively correlated with saturation and bias in base composition but their removal is not sufficient to fully eliminate homoplasy. Removing of the fastest evolving eighth nucleotide category in each of the three dataset resulted in improved support only for nodes moderately affected by homoplasy: the deepest node for introns and more recent nodes for exons and mitochondrial genes. Our study fully support the recognition of the five subordinal clades as described in Carleton and Musser [3] but proposed for the first time new intersubordinal clusterings. The relationship between Myomorpha and Anomaluromorpha appears well supported by the intron data in particular whereas the association between Hystricomorpha and Sciuromorpha is better supported when the data are combined and fast-evolving characters are excluded.

## Methods

### Taxon and gene sampling

All five suborders were comprehensively sampled at the familial level apart from the monophyletic superfamily Muroidea and the suborder Hystricognathi (Additional file 3). Outgroups of successive relatedness were obtained from the order Lagomorpha (3 taxa) and the more distantly related orders Primates (one species) and Cetartiodactyla (3 taxa). The complete matrix represents 30 rodents and 7 outgroups with a low amount of missing data (Additional file 3).

### Sequencing

For the present study, sequencing was performed mostly for the intron fragments (MGF, PRKC, SPTBN and THY). DNA was extracted from ear or liver tissue preserved in ethanol using the QIAamp DNA Mini Kit (QIAGEN Inc.). Extracted DNA was used as template in PCR using primers defined in Matthee et al. [40,46] and Eick et al. [60].

Polymerase chain reaction (PCR) was performed using an initial denaturation of 2 min at 94°C, followed by 35 cycles of 45 seconds denaturation at 94°C, 1 minute annealing at 50°C – 55°C, 1 min extension at 72°C, and a 10 minutes final extension at 72°C. Amplified products were purified with a QIAquick PCR Gel Extraction Kit (QIAGEN Inc.). Sequencing was performed with the ABI Prism Big Dye Terminator Cycle Sequencing Ready Reaction Kit, and analysed on an ABI Prism 310 or 3100 DNA Sequencer (Genetic Analyser Applied Biosystems). Samples were edited using Sequencher 4.6 software (Gene Codes Corporation). Sequences have been deposited at the EMBL databank with accession numbers presented in Additional file 3.

### Alignment, partition and saturation analysis

The mitochondrial cyt*b*, and the two nuclear exons (IRBP and vWF) were aligned by hand with the ED editor of the software MUST [61] by making use of codon alignment (indels were always in multiples of three base pairs long). For the 12S rRNA fragment, alignment was performed based on a collection of rodents previously aligned [21] and a secondary structure model (stems and loops; [62]). Indels were placed preferentially in loop regions. The four intron fragments were aligned with more difficulty because of numerous and sometimes long indels. Alignment was first performed at the familial level by aligning all the well established monophyletic groups using T-coffee (version 5.05; [63]). These files were then combined and further manually aligned across families and orders. Alignment was also compared and optimized following the different criteria outlined previously [48]. Finally, poorly aligned positions were eliminated using the Gblocks program (version 0.91b; [64]) with the following options in effect: half the number of sequences for the minimum number of sequences for a conserved position and for a flank position (parameters 1 and 2); maximum number of contiguous nonconserved positions set to 8 (parameter 3); minimum length of a block after gap cleaning fix to 2 (parameter 4); all gap positions can be selected (parameter 5).

Each gene was partitioned based on its function/structure. Coding genes (cyt*b*, vWF and IRBP) were partitioned according to codon positions whereas the 12s rRNA characters were separated into stems and loops. For MGF, PRKC, SPTBN and THY, the intron and exon parts were identified and treated separately (for details on exon/intron bondaries see [40]). Because the exon sequences of the introns were rather short, the 4 regions were combined and treated as a single unit (no partition).

For each partition, saturation was evaluated graphically following the procedure of Philippe et al. [65]. For each taxon pair, the inferred distance was calculated using the program Treeplot of the MUST package [61] and the maximum likelihood tree (model GTR + I + G with PhyML version 2.4.4; [66]) as reference. The inferred distances were plotted against the observed distances (program Comp_mat of the MUST package). The slope of the regression (S) is an indication of the level of saturation: the closer the slope to zero, the more saturated the data.

### Phylogenetic reconstructions

Phylogenetic trees were constructed using parsimony (MP) and two probabilistic approaches: maximum likelihood (ML) and Bayesian inference (BI). MP and BI analyses were run using PAUP* (version 4b10; [67]) and MrBayes (version 3.1.2; [68]) respectively. ML analyses were performed using PhyML (version 2.4.4; [66]) and RAxML (version 2.2.3; [69]). The latter was used to allow for partitioned likelihood analyses.

With the two probabilistic methods, the choice of an adequate model of sequence evolution remains a crucial issue (see for example [70]). The search for the optimal model, using Modelgenerator (version 82; [71]) indicated that the general time reversible model (GTR) was the optimal model selected for 13 of the 16 partitions tested. Nucleotide heterogeneity of substitution rates, estimated with a gamma distribution (G) was included in all cases whereas a proportion of invariable sites (I) was found appropriate for 9 of the 16 partitions (data not shown). Taking into account that PhyML does not allow data-partitioning and that the model choice is limited in MrBayes and RAxML, the GTR + G model was used in all analyses, with the gamma distribution approximated by 4 categories. An additional proportion of invariable site (I) was also included whenever possible (PhyML and MrBayes).

The search for the MP tree was conducted using the following options: heuristic search using random addition of taxa with 10 replications and TBR branch swapping. Nodal support was obtained with 1000 bootstrap replications (random addition of taxa with one replication). With PhyML, the search for the ML tree was performed on the global dataset and nodal support was assessed with 100 bootstrap replications generated using BIONJ starting trees. Maximum likelihood analyses with partition (MLP) were performed in RAxML program. The GTR + G model (option -m GTRGAMMA) was applied to different partitions (option -q multipleModelFileName), and individual α-shape parameters, GTR-rates and base frequencies were estimated and optimized for each partition individually. Nodal support was assessed with the bootstrap procedure (option -b bootsrapRandomNumberSeed) with 100 replications (option -# numberOfRuns). The program CONSENSE of the PHYLIP package (version 3.6; [72]) was used to compute the consensus tree from the 100 bootstrap replications. Analyses with the software MrBayes were performed on the partitioned data sets as described above with independent model optimizations for each partition. Metropolis-Coupled Markov Chain Monte Carlo (MC^3^) were run twice for 2,000,000 generations (for mitochondrial, exon and intron files) or 5,000,000 generations (for concatenated datasets) independently using 4 chains each (one cold and 3 heated chains) and sampled every 100 generations. The log-likelihood stationarity was estimated graphically from .p files of MrBayes and the burn-in was set to the first 200,000 or 500,000 generations (2000 or 5000 trees discarded).

### Removal of fast-evolving sites

Fast-evolving sites were identified using the discrete gamma rate category to which they belong [73]. We used TREE-PUZZLE (version 5.2; [74]) to compute the most probable assignment of rate categories for each position (option w: mixed rate heterogeneity with one invariable and 8 gamma rates). The analysis is based on the GTR model and the values of the six substitution rates were previously estimated with PhyML. Sites belonging to the eighth discrete gamma rate category represent the most rapidly evolving positions and were removed using the NET program in MUST [61]. The whole dataset was treated in 3 separate concatenated files: mitochondrial (cyt*b *and 12S rRNA), exon (IRBP and vWF) and intron (MGF, PRKC, SPTBN and THY, with exonic regions) files. The estimation of site-specific rates was determined using the tree obtained with the concatenated datasets except that the branching order between the three main rodent clades (E, G and H in Figure 1) were specified as a trifurcation based on their short lengths.

Departure from homogeneity in base composition of each sequence was calculated in TREE-PUZZLE (using a 5% level chi-square test) under the GTR + I + G model (with substitution parameters first estimated with PhyML). The level of saturation of each partition (as estimated by the slope of the linear regression: see above) was compared before and after removal of fast-evolving sites.

### Test of alternative hypotheses

The best topology was compared to several alternative hypotheses using PAML (version 3.15; [75]) and CONSEL (version 0.1i; [76]). Tests were conducted on concatenated datasets (before and after removal of fast-evolving sites) with PAML and the GTR + G model was applied to the different partitions with independent parameter estimations (option G Mgene = 4). Log-likelihoods of site-pattern trees (.lnf file) were then used by CONSEL to calculate the P-values for several statistical tests for which only the AU (approximately unbiased) test, SH (Shimodaira-Hasagawa) test and the PP (approximate Bayesian posterior probability) are presented here.

### Molecular dating

Divergence times were estimated using the relaxed Bayesian molecular clock implemented in MULTIDISTRIBUTE (version 09.25.03; [77]). The software allow for multilocus analyses with autocorrelation of rates. We used the reduced-concatenated dataset partitioned in 8 partitions, including the first and second codon positions for the cyt*b *(the third position was not included because sequences were too divergent for calculating distances with PAML), the entire 12s rRNA, three codon positions for the 2 concatenated exons, and 2 partitions for the intron dataset: the four introns combined and the exonic regions combined. We used the topology obtained with the concatenated-reduced dataset (Figure 1) as input. Model parameters were firstly estimated for each partition with PAML using the F84 substitution model with a five-category gamma distribution. Then, estimation of branch lengths and their variance-covariance matrix were performed with the ESTBRANCHES program. Thirdly, MULTIDIVTIME allows estimating divergence times and their variance and the following priors were used: 100 Myr as a prior expected time between tip and root (approximate age of the eutherian radiation; [58]), 0.0003 (calculated as the median of branch lengths over the 8 resulting trees divided by the root age; see MULTIDIVTIME guidelines) as prior distribution for rate at root node, and 0.5 as the mean of the prior for autocorrelation rate parameter along branches. For all parameters, standard deviation equals the value of the parameter. Markov chain Monte Carlo analyses were run for 1,000,000 generations sampled every 100 generations with a burn-in of the first 10,000 generations.

MULTIDIVTIME allows the incorporation of multiple time constraints as well as their uncertainties. Four calibration points were used: 1) between 28 and 35 Mya (Early Oligocene) for the origin of the Heteromyidae and Geomyidae families [8,57]; 2) 37 Mya as the lower bound for the split between Aplodontidae and Sciuridae [57]; 3) between 28 and 50 Mya for the origin of modern glirid lineages (*Graphiurus *and *Dryomys*; [8,57]); 4) between 49 and 55 Myr (Early Eocene) for the split between ochotonids and leporids [78].

## Authors' contributions

CM and CAM conceived the study, and participated in its design. CM was involved in the sequence generation and alignment, performed phylogenetic and dating analyses and drafted the manuscript. VA participated in the sequence generation, designed Additional file 3 and collected bibliographic references. EF carried out sequencing and contributed to phylogenetic analyses. CAM collected samples, participated in the generation and alignment of sequences and helped to draft the manuscript. All authors read and approved the final manuscript.

## Supplementary Material

Additional file 1 **Intron alignment before Gblocks**. Sequence alignment of each intron before removal of poorly aligned positions by Gblocks is given in nexus format. Click here for file

Additional file 2 **Intron alignment after Gblocks**. Sequence alignment of each intron after removal of poorly aligned positions by Gblocks is given in nexus format. Click here for file

Additional file 3 **Taxon and gene samplings**. The taxonomic arrangement follows Carleton and Musser [3]. When sequences were not available from the same species a chimera has been built between genes of different species and is noted "sp" as species name. For Hystricidae and Physeteridae, a chimera has been built between two genera. MGF: Stem cell factor; PRKC: protein kinase C; SPTBN: β-spectrin non erythrocytic 1; THY: Thyrothropin; vWF: the exon 28 of von Willebrand factor; IRBP: exon one of the interphotoreceptor retinoid-binding protein; CYT*b*: mitochondrial cytochrome *b*; 12S rRNA: mitochondrial 12S ribosomal RNA. • denote sequences that have been obtained for the present study and – means that no sequence is available. Click here for file

Additional file 4 **Supports for suprafamilial groupings according to the three separate (mitochondrial, exon and intron genes) and concatenated (conc) datasets**. R indicates that fast-evolving sites have been removed from the dataset and the numbers of characters analysed is indicated below each dataset name. MLP is the bootstrap support resulting from 100 replications in partitioned maximum likelihood analysis with RaxML, BI is the posterior probability in Bayesian analysis with MrBayes, and MP is the bootstrap support resulting from 1000 replications in maximum parsimony analysis with PAUP (for concatenated datasets only). Relationships supported by concatenation are in bold. When existing, the name of suprafamilial groupings is given into brackets. Click here for file

## References

[B1] Brandt JF Beiträge zur nähern Kenntniss der Säugetiere Russlands Mém Acad Imp Sci St-Pétersbourg 1855 9 1 375

[B2] Simpson GG The principles of classification and a classification of mammals Bull Am Mus Nat Hist 1945 85 1 350

[B3] Carleton MD Musser GG Wilson DE, Reeder DM Order Rodentia Mammal Species of the World: A Taxonomic and Geographic reference 2005 Baltimore: Johns Hopkins University Press 745 752

[B4] Huchon D Chevret P Jordan U Kilpatrick CW Ranwez V Jenkins PD Brosius J Schmitz J Multiple molecular evidences for a living mammalian fossil P Natl Acad Sci USA 2007 104 18 7495 7499 10.1073/pnas.0701289104 PMC1863447 17452635

[B5] Jenkins PD Kilpatrick CW Robinson MF Timmins RJ Morphological and molecular investigations of a new family, genus and species of rodent (Mammalia: Rodentia: Hystricognatha) from Lao PDR Syst Biodivers 2004 2 419 454 10.1017/S1477200004001549

[B6] Wood AE Grades and clades among rodents Evolution 1965 19 115 130 10.2307/2406300

[B7] Hartenberger J-L Luckett WP, Hartenberger J-L The order Rodentia: Major questions on their evolutionary origin, relationships and suprafamily systematics Evolutionary Relationships Among Rodents: a Multidisciplinary Analysis 1985 New York: Plenum Press 1 33

[B8] Hartenberger J-L Description de la radiation des Rodentia (Mammalia) du Paléocène supérieur au Miocène; incidences phylogénétiques C R Acad Sci Paris 1998 326 439 444

[B9] Landry SO A proposal for a new classification and nomenclature for the Glires (Lagomorpha and Rodentia) Mitt Mus Nat Berl Zool Reihe 1999 75 283 316

[B10] Huchon D Catzeflis FM Douzery JPE Variance of molecular datings, evolution of rodents and the phylogenetic affinities between Ctenodactylidae and Hystricognathi Proc R Soc London ser B 2000 267 393 402 10.1098/rspb.2000.1014 PMC1690539 10722222

[B11] Farwick A Jordan U Fuellen G Huchon D Catzeflis F Brosius J Schmitz J Automated scanning for phylogenetically informative transposed elements in Rodents Syst Biol 2006 55 6 936 948 10.1080/10635150601064806 17345675

[B12] Marivaux L Vianey-Liaud M Jaeger JJ High-level phylogeny of early Tertiary rodents: dental evidence Zool J Linn Soc 2004 142 1 105 134 10.1111/j.1096-3642.2004.00131.x

[B13] Waddell PJ Shelley S Evaluating placental inter-ordinal phylogenies with novel sequences including RAG1, gamma-fibrinogen, ND6, and mt-tRNA, plus MCMC-driven nucleotide, amino acid, and codon models Mol Phylogenet Evol 2003 28 2 197 224 10.1016/S1055-7903(03)00115-5 12878459

[B14] Lavocat R Parent J-P Luckett WP, Hartenberger JL Phylogenetic analysis of middle ear features in fossil and living rodents Evolutionary Relationships Among Rodents: a Multidisciplinary Analysis 1985 New York: Plenum press 333 354

[B15] Luckett WP Luckett WP, Hartenberger JL Superordinal and intraordinal affinities of rodents:developemental evidence from the dentition and placentation Evolutionary Relationships Among Rodents: a Multidisciplinary Analysis 1985 New York: Plenum press 227 278

[B16] Vianey-Liaud M Luckett WP, Hartenberger J-L Possible evolutionary relationships among Eocene and lower Oligocene rodents in Asia, Europe and North America Evolutionary Relationships Among Rodents: a Multidisciplinary Analysis 1985 New York: Plenum Press 277 309

[B17] Wahlert JH Luckett WP, Hartenberger J-L Cranial foramina of rodents Evolutionary Relationships Among Rodents: a Multidisciplinary Analysis 1985 New York: Plenum Press 311 332

[B18] Sarich VM Of Molecules, Comparative Anatomy, and the Fossil Record – the Evolutionary Messages Cannot Conflict Am J Phys Anthropol 1985 66 2 224 224

[B19] Adkins RM Walton AH Honeycutt RL Higher-level systematics of rodents and divergence time estimates based on two congruent nuclear genes Mol Phylogenet Evol 2003 26 3 409 420 10.1016/S1055-7903(02)00304-4 12644400

[B20] Huchon D Madsen O Sibbald MJJ Ament K Stanhope MJ Catzeflis FM De Jong WW Douzery JPE Rodent phylogeny and a timescale for the evolution of Glires: evidence from an extensive taxon sampling using three nuclear genes Mol Biol Evol 2002 19 1053 1065 12082125 10.1093/oxfordjournals.molbev.a004164

[B21] Montgelard C Bentz S Tirard C Verneau O Catzeflis FM Molecular systematics of Sciurognathi (Rodentia): the mitochondrial cytochrome b and 12S rRNA genes support the Anomaluroidea (Pedetidae and Anomaluridae) Mol Phylogenet Evol 2002 22 220 233 10.1006/mpev.2001.1056 11820843

[B22] Bugge J The cephalic arterial system in mole-rats (Spalacidae) bamboo rats (Rhizomyidae), jumping mice and jerboas (Dipodoidea) and dormice (Gliroidea) with special reference to the systematic classification of rodents Act Anat 1971 79 2 165 180 10.1159/000143636 5113853

[B23] Nedbal MA Honeycutt RL Schlitter DA Higher-level systematics of rodents (Mammalia, Rodentia): Evidence from the mitochondrial 12S rRNA gene J Mammal Evol 1996 3 3 201 237 10.1007/BF01458181

[B24] Tullberg T Ueber das System der Nagetiere:eine phylogenetische Studie Nova Acta Reg Soc Sci Upsaliensis Ser 3 1899 18 1 514

[B25] Montgelard C Bentz S Douady C Lauquin J Catzeflis FM Molecular phylogeny of the sciurognath families Gliridae, Anomaluridae and Pedetidae: morphological and paleontological implications8th African Small Mammal Symposium: 4–9 Juillet 1999 2001; Paris: IRD 2001 293 307

[B26] Winge H Jordfundne og nulevende Gnavere (Rodentia) fra Lagoa Santa, Mina Gerais, Brasilien E Museo Lundii 1887 1 1 178

[B27] Horner DS Lefkimmiatis K Reyes A Gissi C Saccone C Pesole G Phylogenetic analyses of complete mitochondrial genome sequences suggest a basal divergence of the enigmatic rodent Anomalurus BMC Evol Biol 2007 7 10.1186/1471-2148-7-16 PMC1802082 17288612

[B28] D'Erchia AM Gissi C Pesole G Saccone C Arnason U The guinea-pig is not a rodent Nature 1996 381 597 600 10.1038/381597a0 8637593

[B29] Graur D Hide WA Li WH Is the Guinea-Pig a Rodent Nature 1991 351 6328 649 652 10.1038/351649a0 2052090

[B30] Adkins RM Gelke EL Rowe D Honeycutt RL Molecular phylogeny and divergence time estimates for major rodent groups/evidence from multiple genes Mol Biol Evol 2001 18 777 791 11319262 10.1093/oxfordjournals.molbev.a003860

[B31] Amrine-Madsen H Koepfli KP Wayne RK Springer MS A new phylogenetic marker, apolipoprotein B, provides compelling evidence for eutherian relationships Mol Phylogenet Evol 2003 28 2 225 240 10.1016/S1055-7903(03)00118-0 12878460

[B32] Veniaminova NA Vassetzky NS Kramerov DA B1SINEs in different rodent families Genomics 2007 89 6 678 686 10.1016/j.ygeno.2007.02.007 17433864

[B33] Delsuc F Brinkmann H Philippe H Phylogenomics and the reconstruction of the tree of life Nat Rev Genet 2005 6 5 361 375 10.1038/nrg1603 15861208

[B34] Jeffroy O Brinkmann H Delsuc F Philippe H Phylogenomics: the beginning of incongruence? Trends Genet 2006 22 4 225 231 10.1016/j.tig.2006.02.003 16490279

[B35] Phillips MJ Delsuc F Penny D Genome-scale phylogeny and the detection of systematic biases Mol Biol Evol 2004 21 7 1455 1458 10.1093/molbev/msh137 15084674

[B36] Gruber KF Voss RS Jansa SA Base-compositional heterogeneity in the RAG1 locus among didelphid marsupials: Implications for phylogenetic inference and the evolution of GC content Syst Biol 2007 56 1 83 96 10.1080/10635150601182939 17366139

[B37] Rodriguez-Ezpeleta N Brinkmann H Roure B Lartillot N Lang BF Philippe H Detecting and Overcoming Systematic Errors in Genome-Scale Phylogenies Syst Biol 2007 56 3 389 399 10.1080/10635150701397643 17520503

[B38] Ruano-Rubio V Fares MA Artifactual phylogenies caused by correlated distribution of substitution rates among sites and lineages: The good, the bad, and the ugly Syst Biol 2007 56 1 68 82 10.1080/10635150601175578 17366138

[B39] Pisani D Identifying and removing fast-evolving sites using compatibility analysis: an example from the Arthropoda Syst Biol 2004 53 978 989 10.1080/10635150490888877 15764565

[B40] Matthee CA Burzlaff JD Taylor JF Davis SK Mining the mammalian genome for artiodactyls phylogeny Syst Biol 2001 50 367 390 10.1080/106351501300317987 12116581

[B41] Springer MS DeBry RW Douady C Amrine HM Madsen O de Jong WW Stanhope MJ Mitochondrial versus nuclear gene sequences in deep-level mammalian phylogeny reconstruction Mol Biol Evol 2001 18 2 132 143 11158372 10.1093/oxfordjournals.molbev.a003787

[B42] Björklund M Are third positions really that bad ? A test using vertebrate cytochrome b Cladistics 1999 15 191 197 10.1111/j.1096-0031.1999.tb00261.x 34902919

[B43] Broughton RE Stanley SE Durrett RT Quantification of homoplasy for nucleotide transitions and transversions and a reexamination of assumptions in weighted phylogenetic analysis Syst Biol 2000 49 4 617 627 10.1080/106351500750049734 12116430

[B44] de Queiroz A Gatesy J The supermatrix approach to systematics Trends Ecol Evol 2007 22 34 41 10.1016/j.tree.2007.08.002 17046100

[B45] Gatesy J Baker RH Hidden likelihood support in genomic data: Can forty-five wrongs make a right? Syst Biol 2005 54 3 483 492 10.1080/10635150590945368 16012113

[B46] Matthee CA van Vuuren BJ Bell D Robinson TJ A molecular supermatrix of the rabbits and hares (Leporidae) allows for the identification of five intercontinental exchanges during the Miocene Syst Biol 2004 53 3 433 447 10.1080/10635150490445715 15503672

[B47] Willows-Munro S Robinson TJ Matthee CA Utility of nuclear DNA intron markers at lower taxonomic levels: Phylogenetic resolution among nine Tragelaphus spp Mol Phylogenet Evol 2005 35 3 624 636 10.1016/j.ympev.2005.01.018 15878131

[B48] Matthee CA Eick G Willows-Munro S Montgelard C Pardini AT Robinson TJ Indel evolution of mammalian introns and the utility of non-coding nuclear markers in eutherian phylogenetics Mol Phylogenet Evol 2007 42 827 837 10.1016/j.ympev.2006.10.002 17101283

[B49] Bergsten J A review of long-branch attraction Cladistics 2005 21 163 193 10.1111/j.1096-0031.2005.00059.x 34892859

[B50] Jow H Hudelot C Rattray M Higgs PG Bayesian phylogenetics using an RNA substitution model applied to early mammalian evolution Mol Biol Evol 2002 19 9 1591 1601 12200486 10.1093/oxfordjournals.molbev.a004221

[B51] DeBry RW Identifying conflicting signal in a multigene analysis reveals a highly resolved tree: The phylogeny of Rodentia (Mammalia) Syst Biol 2003 52 5 604 617 10.1080/10635150390235403 14530129

[B52] Hudelot C Gowri-Shankar V Jow H Rattray M Higgs PG RNA-based phylogenetic methods: application to mammalian mitochondrial RNA sequences Mol Phylogenet Evol 2003 28 2 241 252 10.1016/S1055-7903(03)00061-7 12878461

[B53] Kjer KM Honeycutt RL Site specific rates of mitochondrial genomes and the phylogeny of eutheria BMC Evol Biol 2007 7 10.1186/1471-2148-7-8 PMC1796853 17254354

[B54] Murphy WJ Eizirik E O'Brien SJ Madsen O Scally M Douady CJ Teeling E Ryder OA Stanhope MJ de Jong WW Resolution of the early placental mammal radiation using Bayesian phylogenetics Science 2001 294 5550 2348 2351 10.1126/science.1067179 11743200

[B55] Hillis DM SINEs of the perfect character P Natl Acad Sci USA 1999 96 18 9979 9981 10.1073/pnas.96.18.9979 PMC33719 10468541

[B56] Springer MS Murphy WJ Eizirik E O'Brien SJ Placental mammal diversification and the Cretaceous-Tertiary boundary P Natl Acad Sci USA 2003 100 3 1056 1061 10.1073/pnas.0334222100 PMC298725 12552136

[B57] McKenna MC Bell SK Classification of mammals above the species level 1997 Columbia University Press

[B58] Archibald JD Averianov AO Ekdale EG Late Cretaceous relatives of rabbits, rodents, and other extant eutherian mammals Nature 2001 414 6859 62 65 10.1038/35102048 11689942

[B59] Maier W Schrenk F The hystricomorphy of the Bathyergidae, as determined from ontogenic evidence Z Saügetierk 1987 52 3 156 164

[B60] Eick GN Jacobs DS Matthee CA A Nuclear DNA Phylogenetic Perspective on the Evolution of Echolocation and Historical Biogeography of Extant Bats (Chiroptera) Mol Biol Evol 2005 22 9 1869 1886 10.1093/molbev/msi180 15930153

[B61] Philippe H MUST: a computer package of management utilities for sequences and trees Nucleic Acids Res 1993 21 5264 5272 10.1093/nar/21.22.5264 8255784 PMC310646

[B62] Springer MS Douzery E Secondary structure and patterns of evolution among mammalian mitochondrial 12S rRNA molecules J MolEvol 1996 43 4 357 373 10.1007/BF02339010 8798341

[B63] Notredame C Higgins DG Heringa J T-Coffee: A novel method for fast and accurate multiple sequence alignment J Mol Biol 2000 302 1 205 217 10.1006/jmbi.2000.4042 10964570

[B64] Castresana J Selection of conserved blocks from multiple alignments for their use in phylogenetic analysis Mol Biol Evol 2000 17 4 540 552 10742046 10.1093/oxfordjournals.molbev.a026334

[B65] Philippe H Sörhannus U Baroin A Perasso R Gasse F Adoutte A Comparison of molecular and paleontological data in diatoms suggests a major gap in the fossil record J Evol Biol 1994 7 247 265 10.1046/j.1420-9101.1994.7020247.x

[B66] Guindon S Gascuel O A simple, fast, and accurate algorithm to estimate large phylogenies by maximum likelihood Syst Biol 2003 52 5 696 704 10.1080/10635150390235520 14530136

[B67] Swofford DL PAUP*. Phylogenetic Analysis Using Parsimony (* and Other Methods), Version 4 1999 Sunderland, Massachusetts.: Sinauer Associates

[B68] Ronquist F Huelsenbeck JP MrBayes 3: Bayesian phylogenetic inference under mixed models Bioinformatics 2003 19 12 1572 1574 10.1093/bioinformatics/btg180 12912839

[B69] Stamatakis A RAxML-VI-HPC: Maximum likelihood-based phylogenetic analyses with thousands of taxa and mixed models Bioinformatics 2006 22 21 2688 2690 10.1093/bioinformatics/btl446 16928733

[B70] Kelchner SA Thomas MA Model use in phylogenetics: nine key questions Trends Ecol Evol 2007 22 2 87 94 10.1016/j.tree.2006.10.004 17049674

[B71] Keane TM Creevey CJ Pentony MM Naughton TJ Mclnerney JO Assessment of methods for amino acid matrix selection and their use on empirical data shows that ad hoc assumptions for choice of matrix are not justified BMC Evol Biol 2006 6 29 10.1186/1471-2148-6-29 16563161 PMC1435933

[B72] Felsenstein J PHYLIP (Phylogeny Inference Package) version 3.6 2004 Seattle: Department of Genome Sciences, University of Washington

[B73] Burleigh JG Mathews S Phylogenetic signal in nucleotide data from seed plants: Implications for resolving the seed plant tree of life Am J Bot 2004 91 10 1599 1613 10.3732/ajb.91.10.1599 21652311

[B74] Schmidt HA Strimmer K Vingron M von Haeseler A TREE-PUZZLE: maximum likelihood phylogenetic analysis using quartets and parallel computing Bioinformatics 2002 18 3 502 504 10.1093/bioinformatics/18.3.502 11934758

[B75] Yang Z PAML: a program package for phylogenetic analysis by maximum likelihood Comput Appl Biosci 1997 13 555 556 9367129 10.1093/bioinformatics/13.5.555

[B76] Shimodaira H Hasegawa M CONSEL: for assessing the confidence of phylogenetic tree selection Bioinformatics 2001 17 1246 1247 10.1093/bioinformatics/17.12.1246 11751242

[B77] Thorne JL Kishino H Divergence time and evolutionary rate estimation with multilocus data Syst Biol 2002 51 5 689 702 10.1080/10635150290102456 12396584

[B78] Rose KD DeLeon VB Missiaen P Rana RS Sahni A Singh L Smith T Early Eocene lagomorph (Mammalia) from Western India and the early diversification of Lagomorpha Proc R Soc London ser B 2008 275 1639 1203 1208 10.1098/rspb.2007.1661 PMC2602686 18285282

