# Supplementary material for: Suprafamilial relationships among Rodentia and the phylogenetic effect of removing fast-evolving nucleotides in mitochondrial, exon and intron fragments
Source: BMC Evol Biol. 2008 Nov 26;8:321. doi: 10.1186/1471-2148-8-321 (PMC2613922; doi:10.1186/1471-2148-8-321)
Supplement: Additional file 3 — Taxon and gene samplings. The taxonomic arrangement follows Carleton and Musser [3]. When sequences were not available from the same species a chimera has been built between genes of different species and is noted "sp" as species name. For Hystricidae and Physeteridae, a chimera has been built between two genera. MGF: Stem cell factor; PRKC: protein kinase C; SPTBN: β-spectrin non erythrocytic 1; THY: Thyrothropin; vWF: the exon 28 of von Willebrand factor; IRBP: exon one of the interphotoreceptor retinoid-binding protein; CYTb: mitochondrial cytochrome b; 12S rRNA: mitochondrial 12S ribosomal RNA. • denote sequences that have been obtained for the present study and – means that no sequence is available. [file 1471-2148-8-321-S3.pdf]

Additional file 3 – Taxon and gene samplings

The taxonomic arrangement follows Carleton and Musser [3]. When sequences were not available from the same species a chimera has been built between genes of different species and is noted “sp” as species name. For Hystricidae and Physeteridae, a chimera has been built between two genera. MGF: Stem cell factor; PRKC: protein kinase C; SPTBN: β-spectrin non erythrocytic 1; THY: Thyrothropin; vWF: the exon 28 of von Willebrand factor; IRBP: exon one of the interphotoreceptor retinoid-binding protein; CYT*b*: mitochondrial cytochrome *b*; 12S rRNA: mitochondrial 12S ribosomal RNA.

* denote sequences that have been obtained for the present study and – means that no sequence is available

SUBORDER<br>Family<br>*Genus species*	MGF	PRKC	SPTBN	THY	vWF	IRBP	CYTb	12S rRNA
SCIUROMORPHA								
Sciuridae								
Tamiasciurus sp	DQ318969	DQ318975	AJ536396	AJ536366	-	AY227622	AF147643	AY227555
Glaucomys sp	AM909781*	DQ318981	AJ536397	AJ536367	AJ224667	AY227598	AJ389531	AF038020
Aplodontidae								
Aplodontia rufa	AM909782*	DQ318982	AJ536398	AJ536364	AJ224662	AJ427238	AJ389528	AJ389541

Gliridae								
Dryomys nitedula	AM909780*	AM909784*	AJ536388	AJ536360	AJ224666	AJ427236	AJ225116	AJ225119
Graphiurus murinus	AM909779*	AM909783*	AJ536395	AJ536371	-	AY303219	AJ225115	AJ225118
MYOMORPHA								
Muridae								
Mus musculus	DQ318971	DQ318977	AL731792	DQ318962	AJ238390	NM_015745	EF108344	EF108344
Acomys sp	AM909770*	AM909789*	AM909797*	AM909818*	AJ402715	AY326074	X96996	X84387
Cricetidae								
Microtus sp	AM909771*	AM909790*	AM909798*	AM909819*	FM200055*	AY163593	U54488	X99464
Mesocricetus auratus	AM909772*	-	AM909799*	AM909820*	AJ402706	AB164047	AF119265	X84390
Spalacidae								
Spalax sp	AM909773*	AM909791*	AM909800*	AM909821*	U31621	U48589	AJ389537	AJ250357
Dipodidae								
Jaculus jaculus	-	AM909837*	AM909832*	AM909827*	AJ297765	-	AJ416890	AJ416890
Dipus sagitta	AM909768*	AM909787*	AM909809*	AM909825*	AJ224665	AJ427232	AM407909	AJ319807
Allactaga sp	AM909769*	AM909788*	AM909810*	AM909815*	AJ224661	AY326076	AJ389534	AJ389545

Napaeozapus insignis	AM909839*	AM909838*	AM909833*	AM909826*	-	AY326098	AJ389535	AJ389546
Sicista sp	AM909767*	AM909786*	AM909808*	AM909822*	AJ297764	FM200058*	-	-
ANOMALUROMORPHA								
Anomaluridae								
Anomalurus sp	AM909775*	AM909793*	AM909806*	AM909816*	AJ427229	AJ427240	AM159537	AJ389539
Idiurus macrotis	AM909774*	AM909792*	AM909807*	AM909817*	FM200056*	-	AJ389525	AJ389538
Pedetidae								
Pedetes sp	DQ318972	DQ318978	AM909831*	DQ318963	AJ238389	AJ427241	AJ389527	AJ389540
CASTORIMORPHA								
Castoridae								
Castor sp	DQ318970	DQ318976	AM909834*	DQ318961	AJ427228	AJ427239	AJ389529	AJ389542
Geomyidae								
Geomys sp	-	-	AM909804*	-	-	AM407914	AF158693	U67300
Cratogeomys castanops	-	AM909835*	AM909830*	-	-	-	L11902	U67299
Thomomys sp	AM909766*	AM909785*	AM909813*	-	AJ427227	AJ427234	AF215808	AF084289
Heteromyidae								

Dipodomys sp	-	-	AM909805*	-	AJ427226	AJ427233	AF172837	U59173
Heteromys gaumeri	-	AM909836*	AM909803*	AM909823*	-	FM200057*	AJ389536	AJ389547
HYSTRICOMORPHA								
Ctenodactylidae								
Ctenodactylus vali	AM909778*	AM909796*	AM909812*	-	AJ238387	-	AJ389532	AJ389543
Massoutiera mzabi	AM909777*	AM909795*	AM909811*	AM909824*	AJ238388	AJ427242	AJ389533	AJ389544
Thryonomyidae								
Thryonomys sp	DQ318974	DQ318980	AM909801*	DQ318965	AJ224674	AJ427243	AJ301644	AJ301644
Bathyergidae								
Bathyergus sp	AM909840*	DQ318983	AM909828*	DQ318968	AJ238384	AJ427251	AF012241	M63565
Caviidae								
Cavia porcellus	AM909776*	AM909794*	AM909802*	AM909814*	AJ224663	AJ427248	AJ222767	AJ222767
Hystricidae	DQ318973	DQ318979	AM909829*	DQ318964	AJ224675	AJ427245	X70674	U12448
Number of rodent sequences	25	27	30	25	24	26	29	29
**OUTGROUP**								
Lagomorpha								

Oryctolagus cuniculus	AY292741	AY292768	AY292810	AY292665	U31618	Z11812	AJ001588	AJ001588
Lepus sp	AY292756	AY292783	AY292825	AY292680	AJ224669	AJ427250	AJ421471	AJ421471
Ochotona princeps	AY292740	AY292766	AY292809	AY292664	AJ224672	AY057832	AJ537415	AJ537415
Primates								
Homo sapiens	AC024941	AC073288	AC093110	AL109660	M25851	NM_002900	AY714024	EF657704
Cetartiodactyla								
Sus scrofa	AF165604	AF165605	AF165606	AF165609	S78431	U48588	AY337045	AY337045
Bos sp	AF165716	AY029293	AF165718	AF165721	X63820	NM_174164	DQ186266	AY526085
Physeteridae	AF165644	AF165645	AF165646	AF165649	AF108834	U50818	NC_002503	NC_002503

List of chimera:

Acomys sp *: A. airensis* (Cytb) *A. dimidiatus* (MGF, PRKC, SPTBN, THY) *A. cahirinus* (VWF, 12S) *A. spinosissimus* (IRBP)

Allactaga sp*: A. elater* (MGF, PRKC, SPTBN, THY , VWF, CYTb, 12S) *A. sibirica* (IRBP)

Bathyergus sp*: B. genetta* (MGF, PRKC, SPTBN, THY, CYTb, 12S) *B. suillus* (VWF, IRBP)

Bos sp *: Bos taurus (PRKC, VWF, IRBP, CYTb,* 12S rRNA *) Bos indicus (MGF, SPTBN, THY)*

Castor sp*: C. canadensis* (MGF, PRKC, SPTBN, THY, VWF, IRBP) *C. fiber* (CYTb, 12S)

Dipodomys sp*: D. merriami* (SPTBN, VWF, IRBP, CYTb) *D. ordii* (12S)

Geomys sp*: G. bursarius* (SPTBN, CYTb, 12S) *G. breviceps* (IRBP)

Glaucomys sp *: G.* sp (MGF, PRKC, SPTBN, THY) *G. volans* (VWF, IRBP, CYTb, 12S)

Hystricidae : *Hystrix africaeaustralis* (MGF, PRKC, SPTBN, THY, CYTb, 12S) *Trichys fasciculata* (VWF, IRBP)

Lepus sp*: L. capensis* (MGF, PRKC, SPTBN, THY) *L. europeus* (CYTb, 12S) *L. crawshayi* (VWF, IRBP)

Microtus sp *: M. arvalis* (MGF, PRKC, SPTBN, THY, CYTb) *M . nivalis* (12S) *M. sikimensis* (IRBP) *M. agrestis* (VWF)

Pedetes sp*: P. capensis (*MGF, PRKC, SPTBN, THY, 12S) *P. surdaster* (VWF, IRBP, CYTb)

Physeteridae: *Kogia breviceps* (MGF, PRKC, SPTBN, THY) *Physeter catodon* (VWF, IRBP, CYTb, 12S)

Sicista sp*: S. betulina* (MGF, PRKC, SPTBN, THY) *S. kazbegica* (IRBP, VWF)

Spalax sp *: S. ehrenbergi* (CytB, 12S) *S. leucodon* (MGF, PRKC, SPTBN, THY) *S. polonicus* (VWF) *S. zemni* (IRBP)

Tamiasciurus sp *: T. hudsonicus* (MGF, PRKC, IRBP, CYTb, 12S), *T.* sp (SPTBN, THY)

Thomomys sp*: T. bottae* (12S), *T. talpoides* (MGF, PRKC, SPTBN, VWF, IRBP, CYTb)

Thryonomys sp *: T.* sp (MGF, SPTBN, THY), *T. swinderianus* (PRKC, VWF, IRBP, CYTb, 12S)
