# Supplementary material for: Suprafamilial relationships among Rodentia and the phylogenetic effect of removing fast-evolving nucleotides in mitochondrial, exon and intron fragments
Source: BMC Evol Biol. 2008 Nov 26;8:321. doi: 10.1186/1471-2148-8-321 (PMC2613922; doi:10.1186/1471-2148-8-321)
Supplement: Additional file 4 — Supports for suprafamilial groupings according to the three separate (mitochondrial, exon and intron genes) and concatenated (conc) datasets. R indicates that fast-evolving sites have been removed from the dataset and the numbers of characters analysed is indicated below each dataset name. MLP is the bootstrap support resulting from 100 replications in partitioned maximum likelihood analysis with RaxML, BI is the posterior probability in Bayesian analysis with MrBayes, and MP is the bootstrap support resulting from 1000 replications in maximum parsimony analysis with PAUP (for concatenated datasets only). Relationships supported by concatenation are in bold. When existing, the name of suprafamilial groupings is given into brackets. [file 1471-2148-8-321-S4.pdf]

Additional file 4 – Supports for suprafamilial groupings according to the three separate (mitochondrial, exon and intron genes) and concatenated (conc) datasets.

R indicates that fast-evolving sites have been removed from the dataset and the numbers of characters analysed is indicated below each dataset name. MLP is the bootstrap support resulting from 100 replications in partitioned maximum likelihood analysis with RaxML, BI is the posterior probability in Bayesian analysis with MrBayes, and MP is the bootstrap support resulting from 1000 replications in maximum parsimony analysis with PAUP (for concatenated datasets only). Relationships supported by concatenation are in bold. When existing, the name of suprafamilial groupings is given into brackets and the letters correspond to clades labelled on Fig 1.

	MITO<br>2126	R-MITO<br>1861	EXON<br>2571	R-EXON<br>2258	INTRON<br>2897	R-INTRON<br>2361	CONC<br>7594	R-CONC<br>6480
	MLP/BI	MLP/BI	MLP/BI	MLP/BI	MLP/BI	MLP/BI	MLP/BI/MP	MLP/BI/MP
**A - Muridae + Dipodidae [Myomorpha]**	93/1.00	79/-	100/1.00	100/1.00	100/1.00	100/1.00	100/1.00/100	100/1.00/100
**B - Pedetidae + Anomaluridae [Anomaluromorpha]**	100/1.00	1/-	93/1.00	50/0.98	100/1.00	100/1.00	100/1.00/100	100/1.00/100

**C - Geomyoidea + Castoridae [Castorimorpha]**	47/0.93	100/1.00	60/1.00	80/1.00	100/1.00	100/1.00	100/1.00/100	100/1.00/100
**D - Myomorpha + Anomaluromorpha**	10/0.17	-/-	38/-	31/0.7	100/1.00	77/0.79	88/0.93/72	73/0.87/41
D2 - Myomorpha + Castorimorpha	14/0.37	2/-	29/0.70	16/0.2	-/-	20/0.14	12/0.06/26	27/0.07/55
D3 - Anomaluromorpha + Castorimorpha	15/0.39	9/-	-/0.05	1/0.09	-/-	3/0.08	-/-/-	-/-/3
**E – (Myomorpha + Anomalu-romorpha) + Castorimorpha**	27/0.97	40/0.99	60/1.00	84/1.00	100/1.00	100/1.00	100/1.00/98	100/1.00/97
**F – Sciuridae + Aplodontidae [Sciuroidea]**	91/1.00	95/1.00	100/1.00	100/1.00	100/1.00	100/1.00	100/1.00/100	100/1.00/100
**G – Sciuroidea + Gliridae [Sciuromorpha]**	80/0.94	90/0.98	55/0.96	81/0.96	99/1.00	100/1.00	100/1.00/98	100/1.00/84
**H – Hystricognathi + Ctenodac-tylidae [Hystricomorpha]**	81/1.00	78/1.00	100/1.00	100/1.00	100/1.00	100/1.00	100/1.00/100	100/1.00/100
**I – Sciuromorpha +**	5/-	29/-	5/0.08	17/0.09	54/0.69	95/1.00	37/0.23/11	91/1.00/48

**Hystricomorpha**								
I2-Sciuromorpha+(Castorimorpha +(Myomorpha+Anomaluromorpha))	-/-	4/0.33	37/0.74	57/0.81	30/0.28	-/-	36/0.32/78	5/-/21
I3 – Hystricomorpha **+** **(**Castorimorpha + (Myomorpha + Anomaluromorpha))	3/0.48	2/-	16/-	11/0.08	16/-	5/-	27/0.45/9	4/-/15
